# A cohort study of accidents occurring in mentally handicapped patients living in institutions

**DOI:** 10.1186/1744-859X-9-22

**Published:** 2010-05-08

**Authors:** Paul Zubillaga, José Ignacio Emparanza, Blanca Guinea, Francisco Mendizábal, Alfonso Muriel, Montserrat Ruiz, Ana María Sánchez, Fernando Sistiaga, Fernando Viguria

**Affiliations:** 1 Uliazpi, Gipuzkoa, San Sebastián, Spain; 2 Donostia Hospital Gipuzkoa, CASPe, CIBER-ESP, San Sebastián, Spain; 3 Gorabide, Bizkaia, Bilbao, Spain; 4 Hospital Ramón y Cajal, CASPe, CIBER-ESP, Madrid, Spain; 5 Fuentes Blancas, Burgos, Spain

## Abstract

**Background:**

Mentally handicapped patients who require extensive and generalised care and are resident in mental health institutions have certain characteristics that could mean that they suffer certain types of accidents. The aim of this study was to determine the number and type of accident-related injuries in this population in order to design appropriate preventative strategies.

**Methods:**

Accident-related injuries in patients resident in six institutions in the north of Spain were recorded prospectively over a period of 21 months. The characteristics of these injuries were recorded in a database linked to another in which patient data were recorded. A logistic regression model employing the generalized estimating equation (GEE) methodology was employed due to the repetition of patient accidents.

**Results:**

There was one death due to foreign body aspiration into the airways. A total of 1,671 injuries were recorded, 0.5% of which were classified as serious, 10% moderate and 89.5% minor. The serious injuries involved fractures (6) and cuts (2), the moderate injuries mainly cuts (57%), bruising (18%) and sprains (13%), and the minor injuries bruising (40%), cuts (35%) and scratches (20%). Falls were the main cause of these injuries (25.2%). The variables associated with serious accidents were self-harm (OR = 1.18), non-collaborative behaviour (OR = 1.21) and inpatient (OR = 1.37).

**Conclusions:**

Accidents in mentally handicapped patients occur in different ways compared to those in the general population. The majority of injuries found were minor (an average of 0.8 to 3.4 accidents per year), with falls being the most common cause. Patients with behavioural disorders undergoing treatment with neuroleptic agents were found to be a risk group, therefore this finding should be taken into consideration when establishing care groups.

## Background

People with some form of mental handicap (MH) make up 1% to 2% of the population. By definition, all these people need lifelong care, which usually varies in intensity and duration depending on their type and degree of handicap. This care is extensive and generalised for the most dependent groups, in other words it covers all types of activities, including very basic self-care and physical protection. These activities are often undertaken in institutions which complement or replace the care provided by the patient's family. In Spain, these institutions attend to patients with a wide range of clinical presentations whose only common element is a mental handicap.

Certain factors suggest that adults with severe MH may, *a priori*, form a unique group with regard to accidents suffered. Some of these factors could be considered protective, such as lack of, or limited, working life and restricted and protected living environment, whereas others, such as lack of, or limited, sense of danger or self-protective ability, together with physical limitations, would appear to favour accidents. In the specific case of institutionalised patients, one should add the close contact between people with very different behavioural and self-defence characteristics as a further negative factor.

In order to be effective, accident-related injury prevention strategies should be based on appropriately collected and evaluated epidemiological data. The information provided in articles published to date concerning this particular patient population is difficult to analyse due to the wide-ranging criteria used to both select the study groups and collect and interpret the data obtained. For this reason, some authors have emphasised the need to approach this topic using objective criteria that allow the actual scale of the problem to be determined and preventative measures to be proposed [1]. The aim of this study was to assess the number and type of accident-related injuries in a population of adult patients with severe MH who live in specific institutions for the majority of the time and to establish the most appropriate accident prevention strategies.

## Methods

### Study design

An observational, analytical, longitudinal, prospective, repeated measures study involving accidents occurring in six institutions in the north of Spain, all of which belong to the public care network, between 1 January 2007 and 30 September 2008. All have their own psychopedagogical, medical and nursing staff, except for centre 2, which relies on community services. These centres were chosen due to the similarities between the type of patients attended and the type of resources available.

### Inclusion criteria

The study included 476 patients of both sexes with severe (IQ 20-34) or profound MH (IQ <20) older than 18 years of age. Injury was defined as any bodily damage resulting directly or indirectly from an external force which required attention by medical staff, either those belonging to the centre itself or from elsewhere, and which occurred accidentally.

### Data collection

Accident-related data were collected by a designated person in each centre, who also collected information from the medical staff who attended the patient. All information was entered into a database designed for this purpose using FileMaker Pro for Windows (FileMaker, Santa Clara, CA, USA). Patient-related variables were entered into the same database but in a different file linked to the former. This information was obtained from the psychologists and medical staff at each centre. Data for patients who were absent from a centre for a period of 1 month or longer were excluded as it was considered that data provided by the families would not be sufficiently accurate. Injuries resulting from typical stereotypic self-harm behaviour were also excluded, although those injuries that, in the same patients, were not considered typical on the basis of their characteristics or intensity, were included.

### Study variables

The patient-related variables were: sex, age, relationship to the centre (inpatient or outpatient; in other words whether they attended the centre in the morning, afternoon or were resident), mobility (able to walk or not), communication level (verbal or non-verbal), serious sight problems, serious hearing problems, active epilepsy, regularly taking neuroleptic or antidepressant medication, polymedication (taking three or more medications from the following groups simultaneously and continually: antiepileptics, neuroleptics, antidepressants), previous accidents (three or more accidents in the year prior to commencement of the study) and behavioural disorders. The latter was assessed on an individual basis by each centre's psychopedagogical staff using the items specified in section E (behavioural problems) of the Inventory for Client and Agency Planning (ICAP) on the basis of non-sporadic behaviours, in other words those with a score of 2 or higher [2]. At the same time, each case was assessed in terms of autistic behaviour as defined by the *Diagnostic and Statistical Manual of Mental Disorders, fourth edition *(DSM-IV).

The accident-related study variables were: date on which the accident happened, injury type, degree of severity (serious when the patient was hospitalised for more than 24 h, moderate when the injury required medication to be administered or the use of sutures, stitches, casts or immobilisation apparatus, and minor in all other cases), body part involved, how the accident happened, where it happened, shift on which it happened (morning, afternoon, night), type of day (weekday or weekend/holiday).

### Statistical methods used

The variables were described statistically using the most appropriate method for their type and measurement scale: absolute and relative frequencies (%) for qualitative variables and mean and standard deviation for quantitative variables.

The association between the existence of at least one moderate/serious accident versus no accident and the existence of at least one minor accident (only for those centres which noted this information) versus no accident was studied by univariate analysis, and the corresponding odds ratio, confidence interval and statistical significance calculated.

For serious accidents, and taking the accidents themselves as analytical unit, we propose a multivariate logistic regression model for those variables found from the univariate analysis to have *P *< 0.1, using a backward modelling strategy. As some patients had several accidents (or as an individual who has had an accident may be more likely to have more), we performed an additional repeated measures logistic regression analysis using the generalized estimating equation (GEE) methodology [3], which takes into account the data correlation structure for each individual.

Statistical analysis was performed using the SPSS program for Windows (SPSS, Chicago, IL, USA), SYSTAT 9.0 (Systat, Chicago, IL, USA) and STATA 9.1 (Stata, College Station, TX, USA).

### Ethical considerations

This study was approved by the Heads of the respective centres and the representatives of the patients' families.

## Results

### Study patients

In all, 14 patients who started the study died during the 21 months that it lasted. One of these deaths was the direct result of an accident (foreign body aspiration). The remaining deaths occurred due to other causes. A further 34 patients were excluded from the study as they were either moved to a different centre or due to prolonged absence, therefore data for 428 patients were evaluated at the end of the study (see Table 1 for patient characteristics).

**Table 1:** Characteristics of the study population

Characteristic	Centre 1	Centre 2	Centre 3	Centre 4	Centre 5	Centre 6	Total
No. of patients cared for	76	16	80	71	103	82	428

Mean age (SD)	35.9 (6.7)	37.5 (9.7)	45.3 (7.5)	44.3 (6.9)	40.7 (10.7)	43.4 (8.3)	41.7 (8.9)

Sex, M/F	33/43	9/7	48/32	43/28	63/40	52/30	248/180 (42.1)

Inpatients, n (%)	60 (79)	9 (56)	76 (95)	60 (84.5)	93 (90)	68 (83)	366 (85.5)

Verbal communication, n (%)	16 (21)	13 (81)	30 (37.5)	20 (28)	31 (30)	33 (40)	143 (33.4)

Mobility (able/unable to walk)	55/21	14/2	59/21	54/17	73/30	72/10	327/101 (76.4)

Autistic, n (%)	37 (49)	2 (12.5)	22 (27.5)	26 (37)	15 (14.5)	32 (39)	134 (31.3)

Previous accidents, n (%)	19 (25)	9 (56)	50 (62.5)	22 (31)	58 (56)	30 (36.5)	188 (43.9)

Sight problems, n (%)	28 (37)	3 (19)	6 (8)	21(29)	55(53)	15 (18)	128 (29.9)

Hearing problems, n (%)	2 (3)	2 (12.5)	5(6)	4 (6)	9 (9)	6 (7)	28 (6.5)

Epileptic, n (%)	35 (46)	4 (25)	35 (44)	27 (38)	40 (39)	37 (45)	178 (41.6)

Taking neuroleptics, n (%)	22 (29)	5 (31)	26 (32)	44 (62)	36 (35)	44 (54)	177 (41.4)

Taking antidepressants, n (%)	1 (1)	0 (0)	2 (2.5)	4 (6)	23 (22)	9 (11)	39 (9.1)

Polymedicated, n (%)	16 (21)	8 (50)	16 (20)	12 (17)	32 (31)	22 (27)	106 (24.8)

Self-harm, n (%)	28 (37)	3 (19)	12 (15)	13 (18)	36 (35)	21 (26)	113 (26.4)

Disruptive behaviour, n (%)	45(59)	11 (69)	44 (55)	32 (45)	26 (25)	32 (39)	190 (44.4)

Non-collaborative behaviour, n (%)	43 (56.5)	11 (69)	33 (41)	29 (41)	18 (17)	41 (50)	80 (18.7)

Offensive social behaviour, n (%)	10 (13)	4 (25)	12 (15)	14 (20)	6 (6)	34 (41)	175 (40.9)

Destructive behaviour, n (%)	11 (14)	3 (19)	11 (14)	6 (8)	19 (18)	9 (11)	59 (13.8)

Stereotypic behaviour, n (%)	62 (81)	6 (37.5)	27 (34)	38 (53.5)	46 (45)	37 (45)	216 (50.5)

Aggressive behaviour, n (%)	19 (25)	7 (44)	24 (30)	15 (21)	31 (30)	20 (25)	116 (27.1)

Behavioural withdrawal, n (%)	45 (59)	6 (37.5)	35 (44)	35 (49)	15 (14.5)	45 (55)	181 (42.3)

### Accidents

A total of 1,671 accidents were recorded, 8 (0.5%) of which were classified as serious, 166 (10%) as moderate and 1,497 (89.5%) as minor. The number of accidents per patient was 6.2, 5.3, 3.5, 2.4, 2.2 and 1.8 for centres 1-6, respectively. Accident-related injuries as a whole were distributed as follows: 676 wounds (40.5%), 632 bruising (38%), 301 scratches (18%), 21 sprains (1%), 14 fractures (0.8%), 8 burns (0.5%), 4 aspirations of liquid or food (0.2%), 3 dislocations (0.2%) and 2 poisonings (0.1%). The category 'others' included seven bites and three cases of accidental removal of the epigastric tube.

The cause of the accident could not be determined in 429 cases (25.5%). The remaining accidents were distributed as follows: 422 (25.2%) due to falls, 330 (20.2%) due to assaults, 146 (8.7%) due to self-harm, 143 (8.5%) due to collisions, 98 (5.8%) due to epileptic seizures, 47 (2.8%) due to sharp objects, 27 (1.6%) due to crushes, 7 (0.4%) due to fire, hot objects or sunlight, 4 (0.2%) due to aspiration and 2 (0.1%) due to ingestion of medications or toxic substances. One skin injury when putting on incontinence pants, one sprained ankle when playing and five accidental removals of epigastric tube due to abrupt turns or movements should also be included.

The injuries were distributed around the body as follows: 738 (44%) in the limbs, 621 (37%) in the head, 248 (15%) on the trunk, and various body parts in the remaining 64 cases (4%).

The place where the accident occurred could not be determined in 458 cases (27.4%). The majority of the remainder occurred in the day rooms (22.5%), followed by bathrooms (11.9%), bedrooms (8.4%) and when moving from one place to another (7.5%).

The majority of accidents (1,111, 66.5%) occurred on work days, with the remainder (430, 25.7%) occurring at weekends/holidays. The day of the week was not specified in 130 cases (7.8%). Almost half the accidents (759, 45.5%) occurred during the day shift, followed by the afternoon (603, 36%) and night shifts (79, 4.7%); the shift was not specified in 230 cases (13.8%).

The variables associated with minor accidents could only be studied for the subgroup of patients from centres 1 and 5. Relationship with the centre and treatment with neuroleptics were the only variables found to be associated with this type of accident (see Table 2).

**Table 2:** Risk factors (univariate analysis) in moderate/serious and minor accidents

	Minor		Serious	
	
Risk variable	OR	95% CI	OR	95% CI
Sex (male)	1.94	0.72 to 5.27	**2.18**	**1.34 to 3.54**

Age (≥ 40)	2.43	0.77 to 7.72	0.91	0.54 to 1.52

Relationship to centre (inpatient)	**11.33**	**3.91 to 32.81**	**4.73**	**2.59 to 8.64**

Communication (verbal)	0.68	0.24 to 1.94	0.67	0.41 to 1.09

Mobility (able to walk)	0.78	0.28 to 2.19	0.81	0.47 to 1.41

Autistic (yes)	1.49	0.47 to 4.75	1.11	0.67 to 1.85

Previous accidents (yes)	2.10	0.72 to 6.18	**4.63**	**2.75 to 7.79**

Sight problems (yes)	1.09	0.41 to 2.90	**2.32**	**1.28 to 4.20**

Hearing problems (yes)	1.13	0.14 to 9.34	1.26	0.45 to 3.48

Epilepsy (yes)	1.15	0.42 to 3.12	**1.69**	**1.04 to 2.76**

Taking neuroleptics	**9.32**	**1.21 to 71.83**	**2.33**	**1.41 to 3.84**

Taking antidepressants			**3.03**	**1.05 to 8.79**

Polymedicated (yes)	1.94	0.54 to 7.02	**2.46**	**1.32 to 4.60**

Self-harm (yes)	1.50	0.51 to 4.43	**2.51**	**1.37 to 4.60**

Disruptive behaviour (yes)	1.35	0.48 to 3.79	**1.80**	**1.11 to 2.91**

Non-collaborative behaviour (yes)	0.79	0.29 to 2.16	**1.81**	**1.11 to 2.94**

Offensive social behaviour (yes)	1.75	0.22 to 14.06	**2.00**	**1.06 to 3.74**

Destructive behaviour (yes)	1.68	0.37 to 7.74	1.67	0.78 to 3.57

Stereotypic behaviour (yes)	0.74	0.26 to 2.07	**1.89**	**1.17 to 3.02**

Aggressive behaviour (yes)	1.40	0.44 to 4.48	**2.09**	**1.20 to 3.63**

Behavioural withdrawal (yes)	0.46	0.17 to 1.24	0.72	0.45 to 1.16

The minor accidents included here are those recorded in centres 1 and 5. Data in bold, *P *< 0.10.

A total of 12 variables showed a significance of *P *= 0.1 in the univariate analysis using the fact of having had a moderate or serious accident or not as dependent variable (Table 2). These variables, and the two found previously for minor accidents, were chosen for subsequent multivariate analysis. As it is known to be an important predictor of accidents, the 'previous accidents' variable was omitted from the multivariate analysis as it is implied in the analyses used.

The variable that reached statistical significance in the multiple logistic regression analysis (Table 3) were sex (more accidents in men), relationship to the centre (more in inpatients than in outpatients), sight problems (more in those who have it), self-harming nature and non-collaborative behaviour.

**Table 3:** Risk factors (multivariate analysis) for moderate or serious accidents

	Logistic regression		GEE logistic regression	
	
Risk variable	OR	95% CI	OR	95% CI
Sex (male)	2.09	1.16 to 3.76		

Relationship to centre (inpatient)	5.52	2.57 to 11.88	1.37	1.06 to 1.77

Sight problems (yes)	2.42	1.21 to 4.84		

Self-harm (yes)	3.04	1.51 to 6.10	1.18	1.04 to 1.34

Non-collaborative behaviour (yes)	1.79	1.06 to 3.37	1.21	1.06 to 1.38

GEE = generalized estimating equation.

The GEE analysis clearly showed the importance of similar, although not the same, patient-related variables to those obtained using the previous logistic model. The multivariate analysis excluding the 'previous accidents' variable, which is included implicitly in the GEE model, revealed that the relationship with the centre, self-harm and non-collaborative behaviour are all associated with the occurrence of accidents.

## Discussion

The large number of accidents, the predominance of minor accidents and the large disparity between the number of accidents per patient in the different centres, which ranges from 6.2 to 1.8 in the 6 centres studied, are of interest. This latter finding is difficult to explain in centres that, at least nominally, have similar characteristics in terms of numbers of staff and patients in care. It therefore appears logical to ascribe these differences to a data collection bias. The criteria used to define serious (hospitalisation for more than 24 h) and moderate accidents (application of sutures, support bandages or casts, medication) are objective and leave little room for interpretation. This is not the case, however, for minor accidents, where the same degree of objectivity is not present. The same haematoma, for example, may be considered worthy of note in one centre but of little importance in another. This proposal is supported when the number of accidents at each centre is considered in terms of their severity. Thus, major differences can be seen in the number of minor accidents per patient (5.9, 1.4, 1.9, 3.1, 4.8 and 1.9) but not in the number of serious (0.01, 0.06, 0.02, 0.01, 0.02 and 0.02) or moderate accidents (0.3, 0.4, 0.3, 0.5, 0.4 and 0.4). This finding appeared sufficiently important to justify separating the moderate and serious cases from the minor ones when both discussing the results and in their subsequent statistical analysis.

One of the deaths recorded during this study occurred in centre 6 and was found to be due to obstruction of the upper airways upon aspiration of pieces of incontinence pants.

The injury was classified as serious in eight cases. Of these, 6 were bone fractures and 2 serious wounds, which together accounted for a total of 27 days in hospital.

Some of the 166 cases classified as moderate were also fractures (5%), although the majority were wounds (57%), bruising (18%) and sprains (13%). The 14 fractures reported during the 21 months of the study account for 3.3% of the study population, a figure well below those reported by Peabody and Stasikelis (67 fractures in a group of 58 patients over 2 years) [4] and Tannenbaum *et al*. (15.3% per year) [5], but similar to that reported by Wagemans and Cluitmans, who recorded 26 fractures in a group of 338 adult patients of all ages over a period of 33 months [6].

A comparison of these figures with those for the general population is of little significance. The percentage of accidents in the Basque Country Health Survey 2002 for the ages of interest here (25-44 and 45-64 years) is 7.4% and 5.5%, respectively [7], versus 40.6% in our series if only serious and moderate cases are included. However, the percentage of hospital admittances in the same survey is 8.3%, versus 1.9% here, which strongly suggests that the classification criteria for an accident are very different.

The results show that, as well as a history of previous accidents, the risk factors for a moderate or serious accident include being an inpatient, self-harming behaviour and a non-collaborative attitude. Accident-related sight problems lose the statistical significance indicated by the logistic regression analysis when previous accidents are considered (in the GEE analysis), thereby suggesting that the influence of the latter is greater. These results are in agreement with our day-to-day experience, and partially so with those reported by Hsieh *et al.*, Sherrard *et al. *and Konarsky *et al. *[8-10], in that patients with behavioural problems, particularly those treated with neuroleptics, suffer more accidents. In contrast to other reports, however, neither epilepsy [8,9,11,12] nor physical limitations [12,13] are risk factors in our series.

In light of the results discussed above, patients with a non-collaborative attitude, who self-harm and are inpatients should be considered as being at high risk of suffering accidents, a finding which should be taken into account when establishing the composition of care groups and training care staff. Another relevant finding is the importance of falls as a cause of injury and the relative frequency of bone fractures. The former suggests the need to remove, as far as possible, all architectural barriers and obstacles and the latter the need for regular check-ups to detect and/or treat predisposing factors such as vitamin D deficiency, which is commonly found in this type of patient.

The majority of the 1,497 minor injuries consisted of bruising (40%), wounds (35%) or scratches (20%). It is impossible to compare these figures with those for the general population as the vast majority of such injuries are usually considered trivial and therefore do not appear in the statistics. Likewise, an intercentre comparison is also of little use for the reasons outlined above. However, minor injuries are rather specific to the patient group in question and, far from being trivial, form a large part of the centres' internal problems in terms of both interactions between care staff themselves and between care staff and patients' families. Indeed, such injuries can be considered to be characteristic of this patient population. Our findings are not unusual in this respect. Thus, in a group of 140 young adults, half of whom had an MH classified as severe or profound, Spreat and Baker-Potts [13] found 147 cases of bruising, 151 scratches, 78 wounds, 75 grazes and 108 bites, among others, over the space of a year.

As with the moderate/serious accidents, falls headed the list of minor accidents. Our findings concur with those of Hsieh [8], who found that half of accidents in his series involved falls. An analysis of these injuries shows that, as well as being able to walk, the predisposing factors here include being an inpatient and taking neuroleptics. Both moderate/serious and minor accidents are more common in the morning than in the afternoon, and much more than at night, and the locations where day-to-day activities are undertaken, along with the bathroom, are the most common accident sites. Serious accidents most often involve the head, whereas minor accidents most often involve the limbs.

## Conclusions

The accidents that occur in the centres covered by this study show characteristics that differentiate them, in terms of both number and type, from those that occur in the general population. These differences include the fact that serious accidents, in other words those that require hospitalisation, occur 4.4 times less often. In contrast, minor accidents are so common in this group that each patient in care suffers an average of between 0.8 and 3.4 accidents per year. Falls are the most common cause of injury for all accident types.

A history of previous accidents is associated with the risk of new accidents for moderate and serious accidents. The other risk factors include being an inpatient, which is associated with a 40% higher risk (OR = 1.37), self-harm, with an almost 20% higher risk (OR = 1.18) and non-collaborative behaviour, with a similar increase (OR = 1.21)

Patients with behavioural disorders should receive special attention from an accident prevention point of view when establishing care groups and training care givers.

## Competing interests

The authors declare that they have no competing interests.

## Authors' contributions

PZ was responsible for coordinating data collection at the different centres and the subsequent interpretation. BG, FM, MR, AMS, FS and FV were responsible for collecting and interpreting data at their respective centres. JIE and AM were responsible for the statistical analysis and interpretation of the results.

## References

[B1] Sherrard J Ozanne-Smith J Staines C Prevention of unintentional injury to people with intellectual disability: a review of the evidence J Intellect Disabil Res 2004 48 639 645 10.1111/j.1365-2788.2003.00570.x 15357683

[B2] Bruininks RH Hill BK Weatherman RF Woodcock RW ICAP. Inventory for Client and Agency Planning. Examiner's Manual 1986 Allen, TX, USA: DLM Teaching Resources

[B3] Zeger SL Liang KY Longitudinal data analysis for discrete and continuous outcomes Biometrics 1986 42 121 130 10.2307/2531248 3719049

[B4] Peabody TD Stasikelis PJ Fractures in adults at an institution for developmentally disabled Clin Orthp Relat Res 1999 366 217 220 10.1097/00003086-199909000-00028 10627738

[B5] Tannenbaum TN Lipworth L Baker S Risk of fractures in an intermediate care facility for persons with mental retardation Am J Ment Retard 1989 93 257 264 2930660

[B6] Wagemans AMA Cluitmans JJM Falls and fractures: a major health risk for adults with intellectual disabilities in residential settings J Policy Pract Intellect Disabil 2006 3 136 138 10.1111/j.1741-1130.2006.00066.x

[B7] Departamento de Sanidad del Gobierno Vasco Encuesta de Salud 2002 http://www.osasun.ejgv.euskadi.net/r52-20726/es/contenidos/informacion/encuesta_salud/es_4044/encues_salud.html

[B8] Hsieh K Heller T Miller AB Risk factors for injuries and falls among adults with developmental disabilities J Intellect Disabil Res 2001 45 76 82 10.1046/j.1365-2788.2001.00277.x 11168779

[B9] Sherrard J Tonge BJ Ozanne-Smith J Injury risk in young people with intellectual disability J Intellect Disabil Res 2002 46 6 16 10.1046/j.1365-2788.2002.00346.x 11851852

[B10] Konarski EA Sutton K Huffman A Personal characteristics associated with episodes off injury in a residential facility Am J Ment Retard 1997 102 37 44 10.1352/0895-8017(1997)102<0037:PCAWEO>2.0.CO;2 9241406

[B11] Jackson RH Accidents and handicap Dev Med Child Neurol 1983 25 656 659 6226553 10.1111/j.1469-8749.1983.tb13828.x

[B12] Slyter EM Garnick DW Kubisiak JM Bishop CE Gilden DM Hakim RB Injury prevalence among children and adolescents with mental retardation Ment Retard 2006 44 212 223 10.1352/0047-6765(2006)44[212:IPACAA]2.0.CO;2 16677066

[B13] Spreat S Baker-Potts JC Patterns of injury in institutionalized mentally retarded residents Ment Retard 1983 21 23 9 6855568

